# Combined exoscopic and endoscopic oblique approach to parafalx lesions while preserving normal structures via the interhemispheric fissure: How I do it

**DOI:** 10.1007/s00701-025-06529-0

**Published:** 2025-04-23

**Authors:** Toshiaki Inomo, Eiji Ito, Mao Yokota, Tadashi Watanabe

**Affiliations:** Department of Neurosurgery, Aichi Medical University, Aichi, Japan

**Keywords:** Selective interhemispheric oblique approach, Exoscope, Endoscope, Falx meningioma, Frontal sinus, Bridging vein

## Abstract

**Background:**

When treating parafalx lesions, the bridging veins or the frontal sinuses can sometimes obstruct access. Preserving these structures to avoid surgical complications is preferred.

**Methods:**

We described the concept of the selective interhemispheric oblique approach (SIOA), which combines exoscopic and endoscopic visualization with gravity-assisted brain retraction to ensure safe access to the parafalx lesions.

**Conclusions:**

The SIOA is a precise and versatile technique for treating parafalx lesions, particularly in anatomically complex areas. With this approach, it’s possible to mitigate risk of venous injury or sinus opening, avoid the retraction of eloquent areas, and obtain excellent surgical outcomes.

**Supplementary Information:**

The online version contains supplementary material available at 10.1007/s00701-025-06529-0.

## Relevant surgical anatomy

The interhemispheric fissure, a narrow but crucial anatomical corridor, separates the cerebral hemispheres and provides primary access to the parafalx lesions. Central to this region is the falx cerebri, a dural structure defining the medial boundary. The superior sagittal sinus runs along the falx’s superior margin, while the inferior sagittal sinus is at its inferior edge [8]. Bridging veins, which drain the cerebral hemispheres into the superior sagittal sinus, traverse this fissure and show significant anatomical variability [7]. The fissure contains critical neurovascular structures, including the gyrus cinguli, corpus callosum, pericallosal arteries, and the cisterna veli interpositi with the internal cerebral veins [1]. The central sulcus region is particularly complex due to a dense network of bridging veins draining the sensorimotor cortex, increasing the risk of neurological compromise [3]. Venous structures within the interhemispheric fissure vary anatomically by location. Between the coronal suture and the postcentral sulcus, larger and more numerous venous lacunae are present, with up to 73% of the superior anastomotic veins of Trolard draining into them [3].

## Description of the technique

### Preoperative preparation

**Fig. 1:**
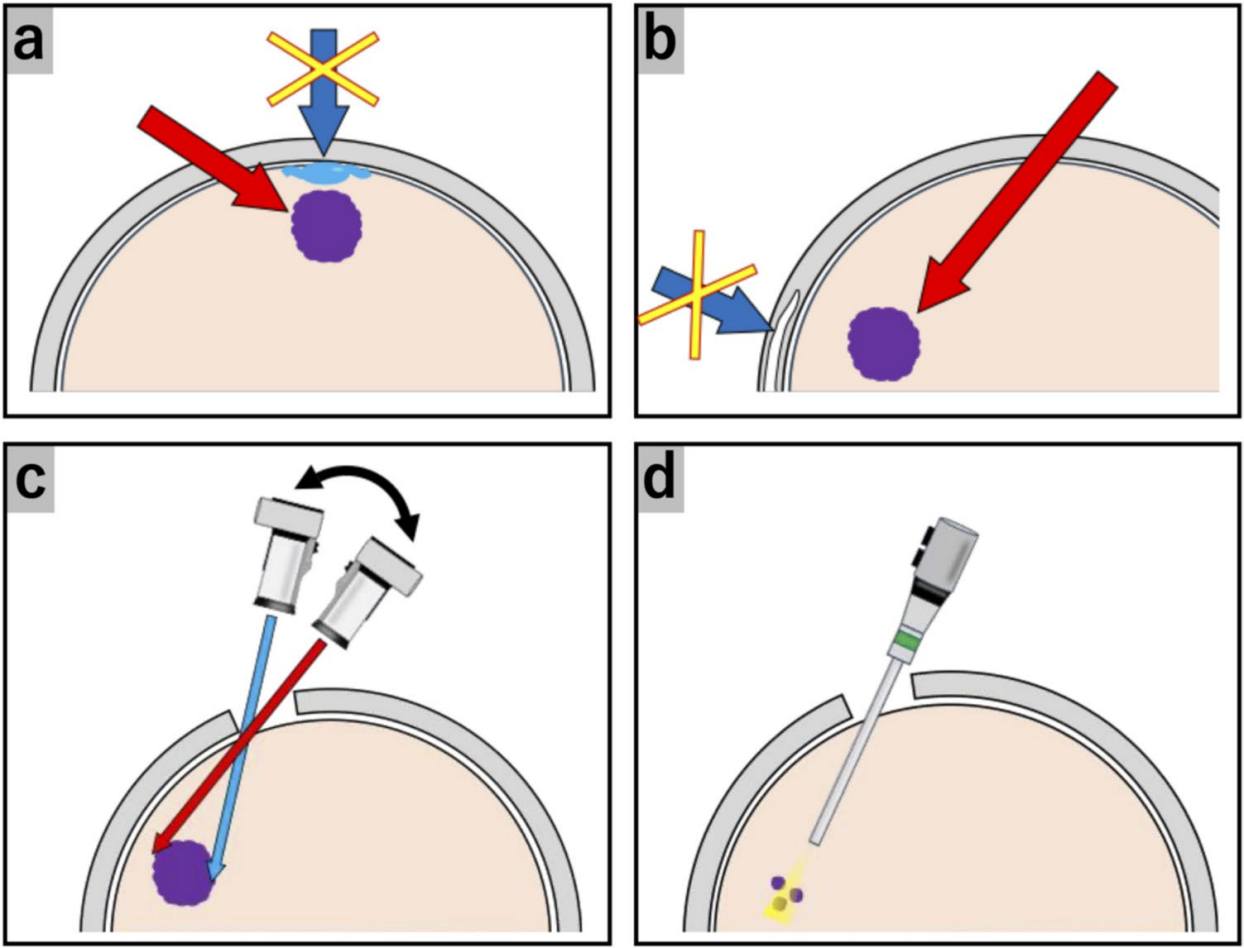
Illustrations of the SIOA concept. **(a)** Anterior oblique approach to preserve normal structures above the lesion, such as bridging veins and eloquent areas. **(b)** Posterior oblique approach to preserve frontal structures, including the frontal sinuses. **(c)** Optimal craniotomy size for visualizing the lesion using an exoscope. **(d)** Use of an endoscope to observe remnant lesions in the exoscope’s blind spots

Preoperative imaging, including computed tomography (CT), three-dimensional CT angiography (3D-CTA), and magnetic resonance imaging (MRI), is essential to assess the lesion’s anatomical relationships with critical structures, including the frontal sinuses, bridging veins, and eloquent areas. To preserve these structures, the optimal entry point/angle and craniotomy size were simulated (Fig. 1a and b). Intraoperative navigation systems (Stealth Station S7; Medtronic, Dublin, Ireland) help to verify these parameters on the scalp surface.

### Surgical position

To leverage gravity for optimal natural brain retraction, the supine lateral position with the affected side down or the supine position with the lesion at the vertex is commonly employed. These positions reduce manual retraction and enhance interhemispheric fissure visualization.

### Surgical technique

A small scalp incision along the hairline enables a tailored craniotomy. The tumor was resected with a 3D exoscope (ORBEYE; Olympus, Tokyo, Japan) for better visualization. A rigid 0° or 30° endoscope (4 mm outer diameter and 18 cm length; Karl Storz, Tuttlingen, Germany) was used as needed to address the exoscope’s blind spots (Fig. 1c and d). After tumor resection, layered wound closure was performed to prevent cerebrospinal fluid (CSF) leakage and maintain cosmetic integrity. The detailed tumor removal procedures are outlined in the following three cases:

#### Case 1: Metastatic frontal lobe tumor near the motor cortex (Fig. 2, Video 1)

**Fig. 2:**
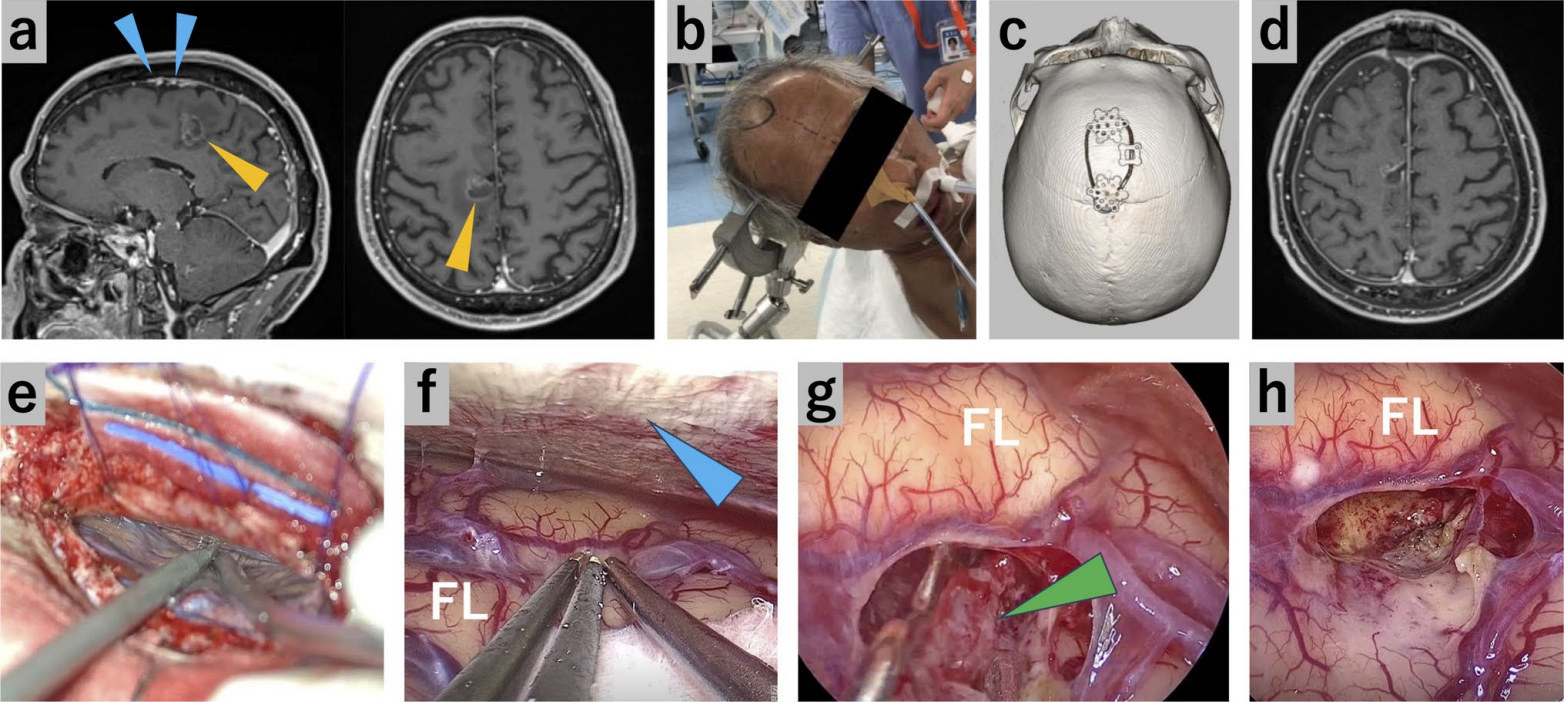
Case 1: Preoperative and postoperative radiologic images, surgical position, and intraoperative exoscopic and endoscopic photographs. (**a**) Preoperative MRI. The sagittal view shows well-developed bridging veins over the tumor. Yellow arrowhead, tumor; blue arrowheads, bridging veins. (**b**) Surgical position, with the head fixed in the supine lateral position and the affected side down. (**c**) Postoperative 3D-CTA, demonstrating craniotomy. (**d**) Postoperative MRI. (**e**) After dural incision, the major bridging veins above the tumor are not seen in the surgical view. (**f**) With CSF drainage, the endoscope is inserted into the interhemispheric fissure. Gravity-assisted brain retraction provides wide surgical fields. Blue arrowhead, the falx cerebri; FL, frontal lobe. (**g**) The tumor is resected under the endoscopic view. Green arrowhead, tumor; FL, frontal lobe. (**h**) After tumor resection. Hemostasis is performed under the endoscopic view. FL, frontal lobe

This was a case of an incidental frontal lobe tumor located in the motor cortex with the bridging veins running over it. The SIOA in the anterior oblique direction was employed for tumor resection to preserve the bridging veins and minimize stress on the motor cortex. Gravity-assisted brain retraction allowed creation of a natural corridor through the interhemispheric fissure, helping complete tumor resection without postoperative neurological deficits.

#### Case 2: Frontal falx meningioma located behind the frontal sinus (Figs. 3 and 4, Video 2)

**Fig. 3:**
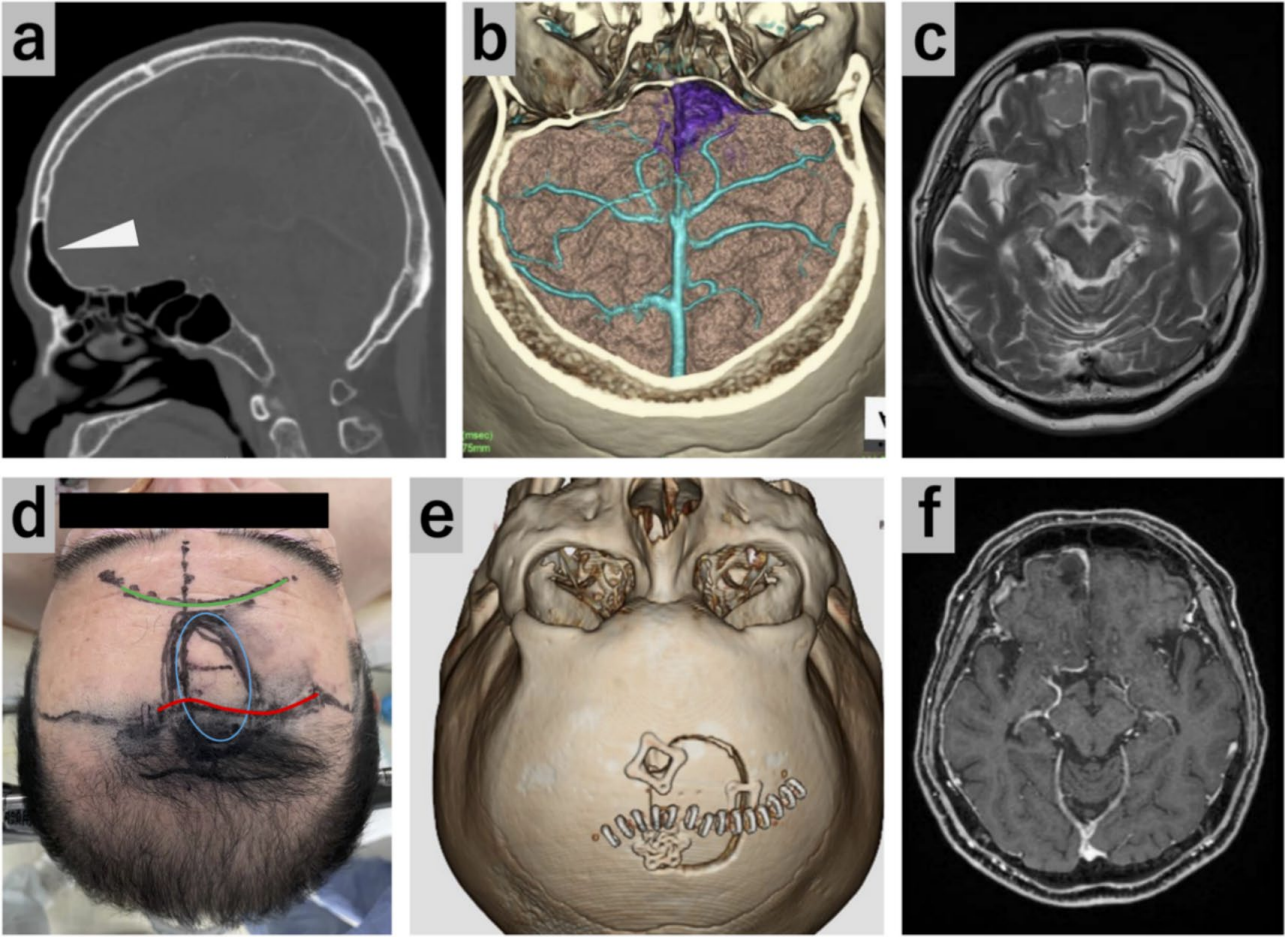
Case 2: Preoperative and postoperative images and surgical position. (**a**) Preoperative sagittal CT showing the developed frontal sinus. White arrowhead, frontal sinus. (**b**) Preoperative 3D-CTA demonstrating major bridging veins in the frontal lobe. The tumor is purple-highlighted. (**c**) Preoperative MRI. (**d**) The surgical position, with the head fixed in the midline position. Green line, top edge of the frontal sinus; red line, skin incision; blue circle, craniotomy size. (**e**) Postoperative 3D-CTA confirming that the craniotomy was performed as simulated. (**f**) Postoperative MRI

**Fig. 4:**
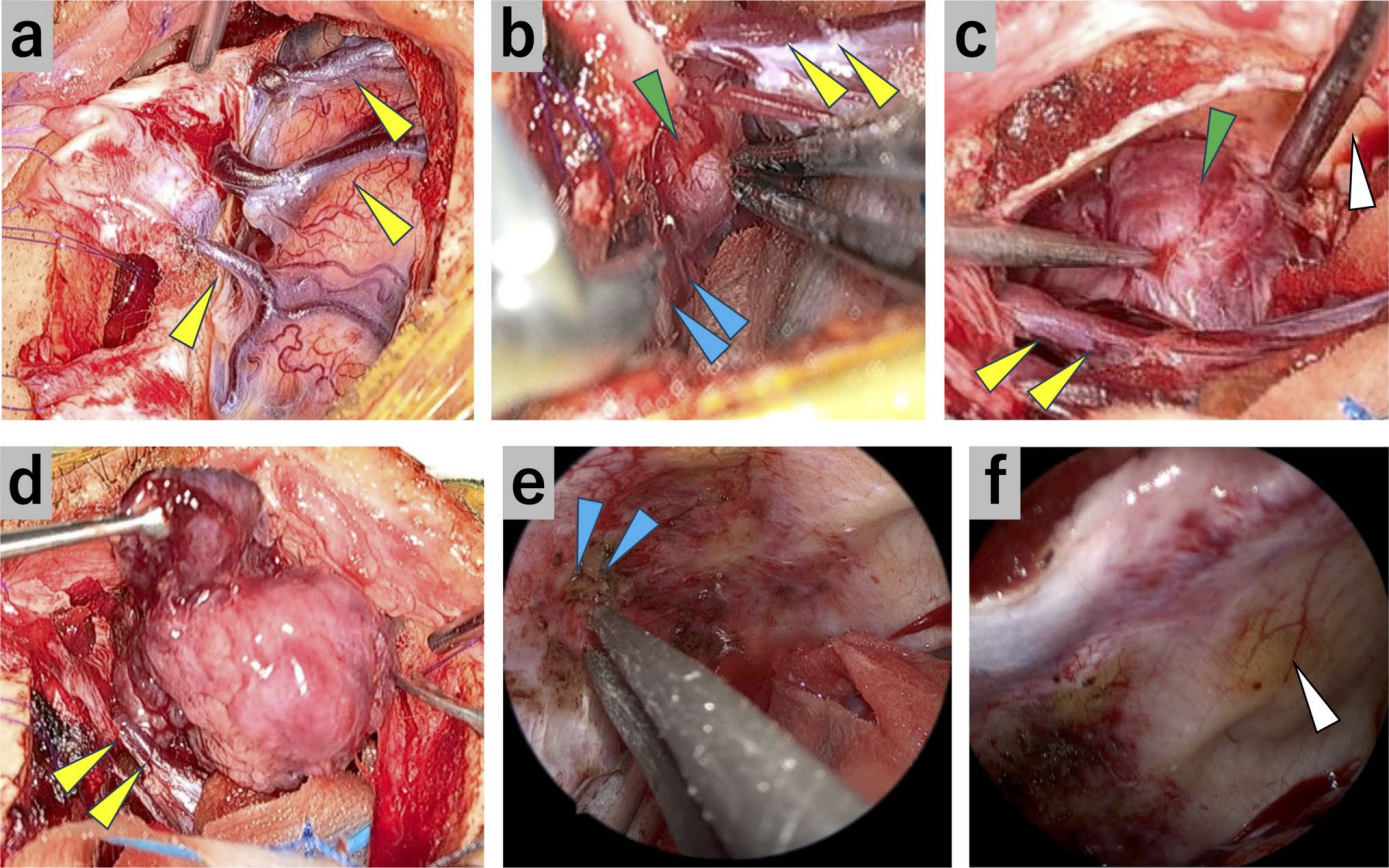
Case 2: Intraoperative exoscopic and endoscopic photographs. (**a**) After dural incision, major bridging veins were present, as observed on preoperative CTA. Yellow arrowheads, bridging veins. (**b**) The tumor’s posterior part is detached from the falx cerebri in the posterior oblique direction. Yellow arrowheads, bridging veins; blue arrowheads, falx cerebri; green arrowhead, tumor. (**c**) With CSF drainage, gravity-assisted brain retraction allowed the observation of the tumor from the frontal space. Yellow arrowheads, bridging veins; green arrowhead, tumor; white arrowhead, the frontal sinus’s posterior wall. (**d**) The tumor is removed *en bloc* through the frontal space. Yellow arrowheads, bridging veins. (**e**) The remnant tumor was resected endoscopically. Blue arrowheads; the falx cerebri. (**f**) After tumor resection. White arrow head; the frontal sinus’s posterior wall

This was a case of frontal falx meningioma identified during a medical checkup. The tumor had to be resected because of its rapid growth. It was located behind the frontal sinus. Several major bridging veins were present over the ipsilateral frontal lobe. The SIOA in the posterior oblique direction was employed for tumor resection to avoid opening the frontal sinus and damaging the bridging veins. Gravity-assisted brain retraction and endoscopic visualization enabled complete tumor resection.

Savardekar et al. reported that the incidence of venous injury during neurosurgical procedures ranges from 2.6% to 30%, with catastrophic complications occurring in a minority of patients [5]. An oblique approach through the subfrontal space or the interhemispheric fissure, aiming to preserve normal structures, is an option that has previously been reported [2, 6]. In addition, gravity-assisted brain retraction is effective for tumor resection, especially for midline lesions [4]. The SIOA combines these techniques and can provide safe and effective resection.

The SIOA incorporates techniques and tools for the microscopic interhemispheric approach, making it applicable to other neurosurgical institutions. The interhemispheric fissure extends in the anteroposterior direction, and by utilizing gravity to retract the brain, additional space is created in the lateral direction, thereby providing a wider operative field. Although internal decompression and frequent adjustments of the visual axis may enable total tumor resection using only an exoscope or microscope, an endoscope helps to ensure complete resection. We use an exoscope at our institution for ergonomical and educational reasons; however it has some drawbacks. First, it requires proper monitor placement, considering the patient’s and the surgical team’s positions. An inappropriate monitor position may lead to ergonomic discomfort, increased fatigue, and a mismatch between vision and operation. Additionally, since the exoscope provides a digital image, the color contrast may be reduced, potentially affecting delicate surgical procedures. Based on these considerations, selection between an exoscope and a microscope should be based on the surgical situation and the surgeon’s experience.

## Indications

The SIOA is primarily recommended for treating parafalx lesions, such as falx meningiomas and medial lesions located within the brain parenchyma. Additionally, its effectiveness also extends to lesions in the medial portion of the anterior cranial fossa, as in Case 2.

## Limitations

The SIOA is not ideal for lesions that compromise the space of the interhemispheric fissure, such as severe brain swelling or massive tumors. For such situations, other surgical techniques like transcortical or basal interhemispheric approaches may be more suitable. Additionally, because the approach route becomes somewhat distant in the SIOA, hemostasis can be challenging in cases of highly vascularized tumors. Although preoperative embolization could potentially address this issue, we currently have insufficient experience and require additional research.

## How to avoid complications

A thorough preoperative assessment of the anatomical structures is crucial. Equally instrumental is the precise simulation of the entry point, entry direction, and craniotomy size. The combination of preoperative imaging (CT, 3D-CTA, and MRI) along with an intraoperative navigation system is more effective. The risk of tumor bleeding can be assessed with contrast-enhanced CT, although angiography provides a more detailed evaluation. To avoid excessive brain retraction and venous injury, a minimal craniotomy size and gravity-assisted techniques are ideal. In addition, meticulous hemostasis and dural closure are crucial for avoiding postoperative bleeding and CSF leakage.

## Specific information for the patient

The general surgical risks involved, such as infection and bleeding, are anticipated. Adequate CSF suction is required to utilize brain retraction by gravity. Therefore, postoperative subdural hematoma is also a concern, especially in the elderly and people with cerebral atrophy.

## 10 key point summary

1. The SIOA is a minimally invasive surgical method for treating lesions in an oblique direction.
2. The SIOA is primarily indicated for lesions around the falx cerebri, such as meningiomas and medial lesions located within the brain parenchyma.
3. Preoperative assessment was conducted to determine the position of the lesion in relation to the critical anatomical structures, including the frontal sinuses, bridging veins, and eloquent areas.
4. An appropriate surgical position was selected for each patient to leverage gravity for effective brain retraction.
5. A lazy S- or Z-shaped skin incision was made along the hairline, followed by craniotomy.
6. The lesion was approached obliquely via the interhemispheric fissure.
7. Under the exoscopic view, the lesion was resected to the greatest extent possible.
8. Internal decompression and frequent adjustments of the visual axis may be beneficial, depending on the circumstances.
9. The endoscope aids in removing any residual tumor in the blind spots on the exoscope.
10. Layered wound closure prevented CSF leakage and preserved the patient’s cosmetic appearance.

## Supplementary Information

Below is the link to the electronic supplementary material.

Supplementary file1 (MP4 235 MB)

Supplementary file2 (MP4 403 MB)

## Data Availability

No datasets were generated or analysed during the current study.

## Abbreviations

*SIOA*: Selective interhemispheric oblique approach
*CT*: Computed tomography
*3D*: Three-dimensional
*MRI*: Magnetic resonance imaging
*CSF*: Cerebrospinal fluid
